# Effects of temporal correlations in social multiplex networks

**DOI:** 10.1038/s41598-017-07591-0

**Published:** 2017-08-17

**Authors:** Michele Starnini, Andrea Baronchelli, Romualdo Pastor-Satorras

**Affiliations:** 1 Departament de Física Fonamental, Universitat de Barcelona, Martí i Franquès 1, 08028 Barcelona, Spain; 2 Universitat de Barcelona Institute of Complex Systems (UBICS), Universitat de Barcelona, Barcelona, Spain; 3 Department of Mathematics - City, University of London - Northampton Square, London, EC1V 0HB UK; 4 Departament de Física, Universitat Politècnica de Catalunya, Campus Nord B4, 08034 Barcelona, Spain

## Abstract

Multi-layered networks represent a major advance in the description of natural complex systems, and their study has shed light on new physical phenomena. Despite its importance, however, the role of the temporal dimension in their structure and function has not been investigated in much detail so far. Here we study the temporal correlations between layers exhibited by real social multiplex networks. At a basic level, the presence of such correlations implies a certain degree of predictability in the contact pattern, as we quantify by an extension of the entropy and mutual information analyses proposed for the single-layer case. At a different level, we demonstrate that temporal correlations are a signature of a ‘multitasking’ behavior of network agents, characterized by a higher level of switching between different social activities than expected in a uncorrelated pattern. Moreover, temporal correlations significantly affect the dynamics of coupled epidemic processes unfolding on the network. Our work opens the way for the systematic study of temporal multiplex networks and we anticipate it will be of interest to researchers in a broad array of fields.

## Introduction

Recently, the theoretical framework of network science^1^ has been enriched by two new concepts: *Multiplex networks* ^2–4^, whose edges belong to different layers, representing different kinds of interactions; and *temporal networks* ^5, 6^, whose edges have an intrinsic dynamics of creation and annihilation, representing interactions switching on and off with given characteristic time scales. The introduction of these two viewpoints has greatly enriched our understanding of real networks. On the one hand, the multiplex representation, through the definition of new observables, such as multilayer clustering, degree correlations or layer overlap^2^, has allowed for a better structural characterization of many networked systems, and helped clarify the behavior of dynamical processes on top of them^7–11^. On the other hand, taking into account the temporal dimension of edges has allowed to uncover unexpected properties of time-varying networks, such as their general bursty nature, characterized by a heavy-tailed distribution of inter-event times *τ* between the establishment of consecutive connections^12, 13^, often compatible with power-law forms, $\psi (\tau ) \sim {\tau }^{-\mathrm{(1}+\alpha )}$. These temporal effects, moreover, have been shown to radically alter the behavior of dynamical processes on such evolving structures^14–17^.

In the particular case of social networks^18^, the recent availability of large digital databases has shown the necessity of a dual description based on both multiplex and temporal network approaches. This urgency stems from the very nature of social interactions, which are diverse in nature and quality, with different layers co-existing and interacting with one another (e.g., physical vs. digital interactions)^19^, and evolve in time, with new relationships being continuously created and destroyed. Therefore, a realistic description should rely on *temporal multiplex networks*, that can be mathematically described by endowing the multiplex paradigm with an additional temporal dimension, see Methods. The empirical evidence of this dual nature of social networks is arousing a growing interest in their temporal multiplex representation within the complex system community^20–22^. However, the effects of the interplay between temporal and multiplex dimensions on the structure and function of real networks still remain largely unexplored, also due to the lack of suitable, longitudinal data.

In this paper, we will consider one particular aspect, namely the possibility of observing correlations between the temporal activity of different layers. In single-layered networks, indeed, temporal correlations have been recently observed^23^, implying the presence of memory effects^24^. In the context of temporal multiplex networks, this effect translates into the possible presence of *inter-layer temporal correlations*, i.e. the fact that a social interaction, taking place in some given layer at some given time, might alter the probability of subsequent interactions in different layers. Such correlations have been characterized in ref. 20 in terms of a Multiplex Markov chain, showing the presence of correlated creation and destruction of connections between pairs of nodes in different layers. Here we focus on the effects of such temporal correlations, both in the dynamics of social interactions and on dynamical processes running on top of a temporal multiplex. We start by checking the presence of inter-layer temporal correlations in several empirical scenarios, by applying a simple information theory approach, which reveals a certain degree of potential predictability in the interaction patterns. We measure the effect of temporal correlations on social activity by defining a multitasking index, and show that they tend to increase the rate of switching between layers expected in an uncorrelated setting. Finally, we explore the impact of temporal correlations on the dynamics of coupled epidemic/awareness processes unfolding on different layers^25^, showing that they can either slow down or speed up the epidemic spread, depending on the region of the parameter space defining the model.

In our analysis, we consider different empirical scenarios: Human contact networks, recorded by the “Reality Mining” (RM) experiment^26^ and consisting of two independent data sets, “Social Evolution” (SE) and “Friends and Family” (FF); Open Source Software (OSS) collaboration networks^27^, with data provided by a OSS project part of the Apache software foundation^28^; and scientific collaboration networks^29^, reconstructed from the American Physical Society (APS) data sets for research^30^. In all cases, interactions are represented as a temporal multiplex network formed by two layers, arbitrarily denoted $\ell \mathrm{=1}$ and $\ell =-1$. See Methods and Section 1 of the Supplementary Material for a full description of the considered data sets.

## Results

### Correlation and influence between layers

One simple approach to establish the presence of inter-layer temporal correlations in our empirical datasets consists in extending to multiplex networks the mutual information analysis traditionally used to detect temporal correlations in single layer sequences of social activity^31–33^. In multiplex networks, an individual *i* switching from one kind of interaction to another one (e.g. he/she sends an email to a colleague and then co-edits some code with another collaborator) is represented by a link between node *i* and node *j* in layer $\ell$ at time *t*_1_ and a link between node *i* and node *k* (including the case *j* = *k*) in layer $-\ell$ at time *t*_2_ > *t*_1_. We want to understand whether *i*, after having an interaction with *j* in layer $\ell$, chooses his next partner *k* in layer $-\ell$ at random or there is a certain degree of predictability in his choice^31, 32^.

To address this issue, we define the uncorrelated entropy ${H}_{i}^{u}(\ell )$ of individual *i* as

1 $${H}_{i}^{u}(\ell )=-\sum _{{j}_{\ell }}{p}_{i}({j}_{\ell })\mathrm{ln}[{p}_{i}({j}_{\ell })],$$

where ${p}_{i}({j}_{\ell })$ is the probability that individual *i* interacts with individual *j* in layer $\ell$. The uncorrelated entropy thus measures the degree of heterogeneity in the interaction pattern of an individual in one layer. The conditional entropy ${H}_{i}^{c}(\ell \to -\ell )$ of individual *i* from layer $\ell$ to layer $-\ell$ is defined as

2 $${H}_{i}^{c}(\ell \to -\ell )=-\sum _{{j}_{\ell }}{p}_{i}({j}_{\ell })\sum _{{k}_{-\ell }}{p}_{i}({k}_{-\ell }|{j}_{\ell })\mathrm{ln}[{p}_{i}({k}_{-\ell }|{j}_{\ell })],$$

where ${p}_{i}({k}_{-\ell }|{j}_{\ell })$ is the conditional probability that individual *i* interacts with individual *k* in layer $-\ell$ immediately after interacting with individual *j* in layer $\ell$. The influence of layer $\ell$ on layer $-\ell$ is quantified by the mutual information, defined for each indiv*i*dual *i* as the difference between uncorrelated and conditional entropy, ${I}_{i}(\ell \to -\ell )={H}_{i}^{u}(-\ell )-{H}_{i}^{c}(\ell \to -\ell )$, thus

3 $${I}_{i}(\ell \to -\ell )=\sum _{{j}_{\ell },{k}_{-\ell }}{p}_{i}({j}_{\ell },{k}_{-\ell })\mathrm{ln}(\frac{{p}_{i}({j}_{\ell },{k}_{-\ell })}{{p}_{i}({j}_{\ell }){p}_{i}({k}_{-\ell })}),$$

where ${p}_{i}({k}_{-\ell },{j}_{\ell })$ is the joint probability that individual *i* interacts first with individual *k* in a layer $-\ell$ and immediately after with individual *j* in a layer $\ell$. Since ${H}_{i}^{u}\ge {H}_{i}^{c}$, the mutual information *I*_*i*_ is always positive, and it is equal to zero only if the interaction patterns of individual *i* on the two layers $-\ell$ and $\ell$ are temporally uncorrelated. Therefore, ${I}_{i}(\ell \to -\ell )$ measures the degree of potential predictability of the interaction pattern of individual *i* in layer $-\ell$, and it is equal to the amount of information about his next partner in layer $-\ell$ earned by knowing his current partner in layer $\ell$.

To avoid spurious effects due sample size issues, in the computation of these quantities we perform a bootstrap analysis, retaining only those individuals who have a value of the conditional entropy significantly smaller than the one obtained by rewiring the network according to a null model, in which, for each individual *i*, the set of all pairs of consecutive interactions in different layers is extracted, and the set of second individual interactions in each pair is randomized. This procedure destroys any temporal correlations between layers, while keeping constant the uncorrelated entropy. See Section 2 of the Supplementary Material for further details.

**Figure 1:**
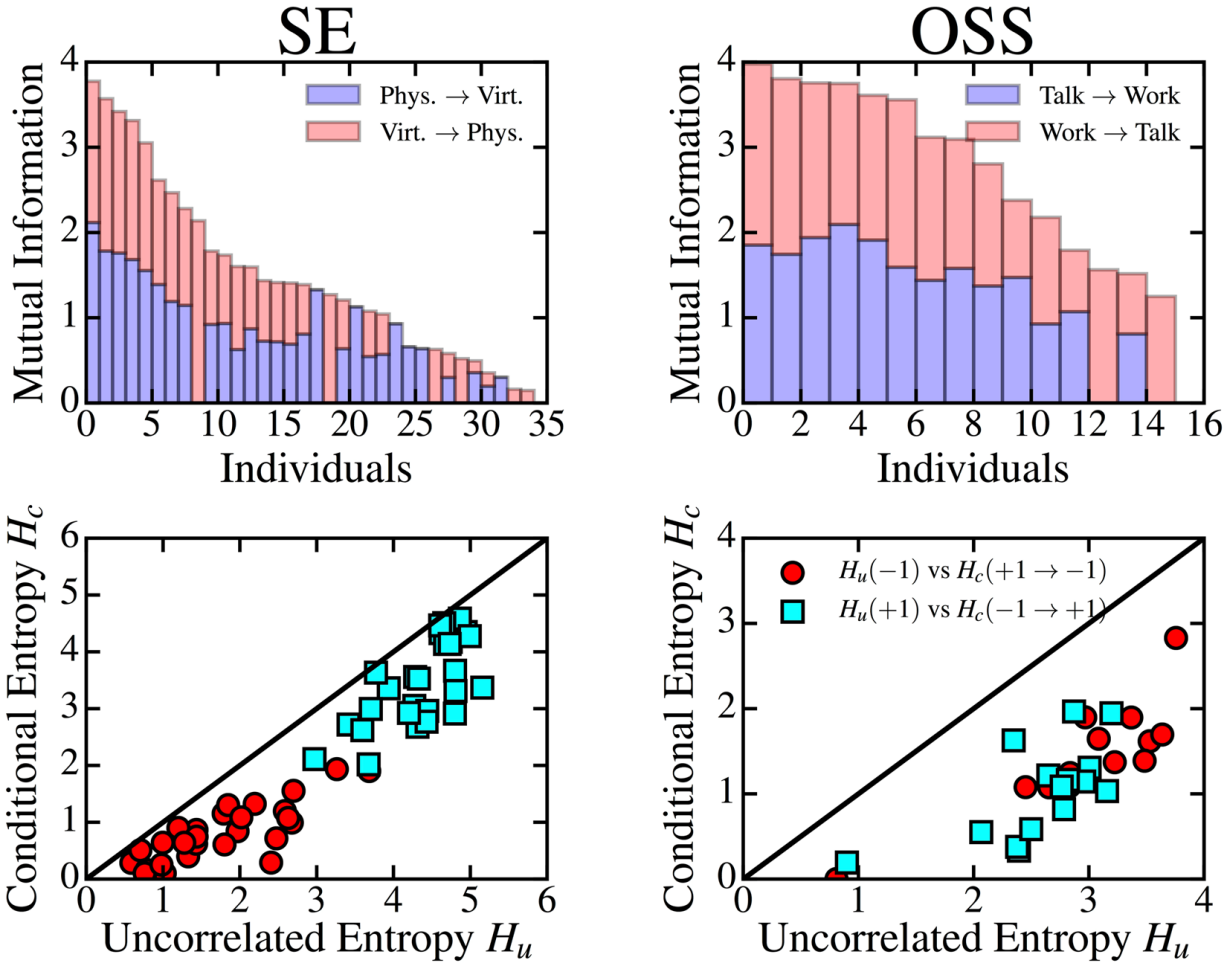
Scatter plot of uncorrelated vs conditional entropy of each individual *i*, ${H}_{i}^{u}(\ell )$ vs ${H}_{i}^{c}(\ell \to -\ell )$ (bottom row), and mutual information between layers, ${I}_{i}(\ell \to -\ell )$ (top row). Only individuals with a conditional entropy with a *p*-value smaller than 0.05 with respect to the null model are plotted. Data shown are: RM contact network, data set SE (left) and OSS collaboration network (right).

Figure 1 (bottom panels) shows the relation between uncorrelated and conditional entropy for single individuals, on the SE contact and OSS collaboration networks (see Supplementary Fig. S2 for additional datasets). One can see that many individuals show a significant entropy difference, resulting in a certain degree of potential predictability, in each data set under consideration. For the case of RM contact networks, in both data sets SE and FF, the uncorrelated and conditional entropy obtained in the physical layer $(\ell =+1)$ are larger than the ones obtained in the digital layer $(\ell =-1)$, because the former is characterized by a richer pattern of interactions, with a larger density and heterogeneity (see Supplementary Table S1). The same behavior is observed in the OSS collaboration network, where the denser communication layer $(\ell =-1)$ shows larger values of the uncorrelated and conditional entropy than the ones obtained in the co-work layer $(\ell =+1)$. Figure 1 (top panels), see also Supplementary Fig. S2, shows the amount of potential predictability of an individual *i* in one layer $\ell$ obtained by the other layer $-\ell$, as measured by mutual information ${I}_{i}(\ell \to -\ell )$. For the majority of individuals there is a mutual influence between layers, both *I*(+1 → −1) > 0 and *I*(−1 → +1) > 0.

These results show, in agreement with ref. 20, that the sequences of contacts in social multiplex networks present indeed temporal correlations. In our mutual information approach, these correlations translate in a certain degree of predictability^31, 32^, resulting from a deterministic component that overrules the random establishment of contacts in one or another social layer. In the following we will show how these temporal correlations can have an impact on social behavior and dynamic spreading.

### Multitasking index of individuals

The temporal correlations observed in the entropy analysis performed above have an effect in the patterns of social interactions that can be gauged by using simple observables. Considering the number of interactions, ${n}_{\ell }^{(i)}({\rm{\Delta }}t)$ and ${n}_{-\ell }^{(i)}({\rm{\Delta }}t)$, that an individual *i* performs in a time interval Δ*t* in two different layers $\ell$ and $-\ell$, a *multitasking index r*_*i*_(Δ*t*) of individual *i* can be defined as the Pearson correlation coefficient between the set of variables $\{{n}_{\ell }^{(i)}({\rm{\Delta }}t),{n}_{-\ell }^{(i)}({\rm{\Delta }}t)\}$, where each pair $({n}_{\ell }({\rm{\Delta }}t),{n}_{-\ell }({\rm{\Delta }}t))$ is measured at different time intervals of fixed length Δ*t*. If *r*_*i*_(Δ*t*) > 0 (i.e. if the values of ${n}_{\ell }^{(i)}({\rm{\Delta }}t)$ and ${n}_{-\ell }^{(i)}({\rm{\Delta }}t)$ attain comparable values), then individual *i* is simply distributing his activity among the two layers and he/she is likely to interact indistinctly in both layers at the same time. Otherwise, if *r*_*i*_(Δ*t*) < 0 (i.e. if a large ${n}_{\ell }^{(i)}({\rm{\Delta }}t)$ is associated with a small ${n}_{-\ell }^{(i)}(\Delta t)$, and vice-versa), then he/she is likely to be concentrating her activity on one of the two layers.

**Figure 2:**
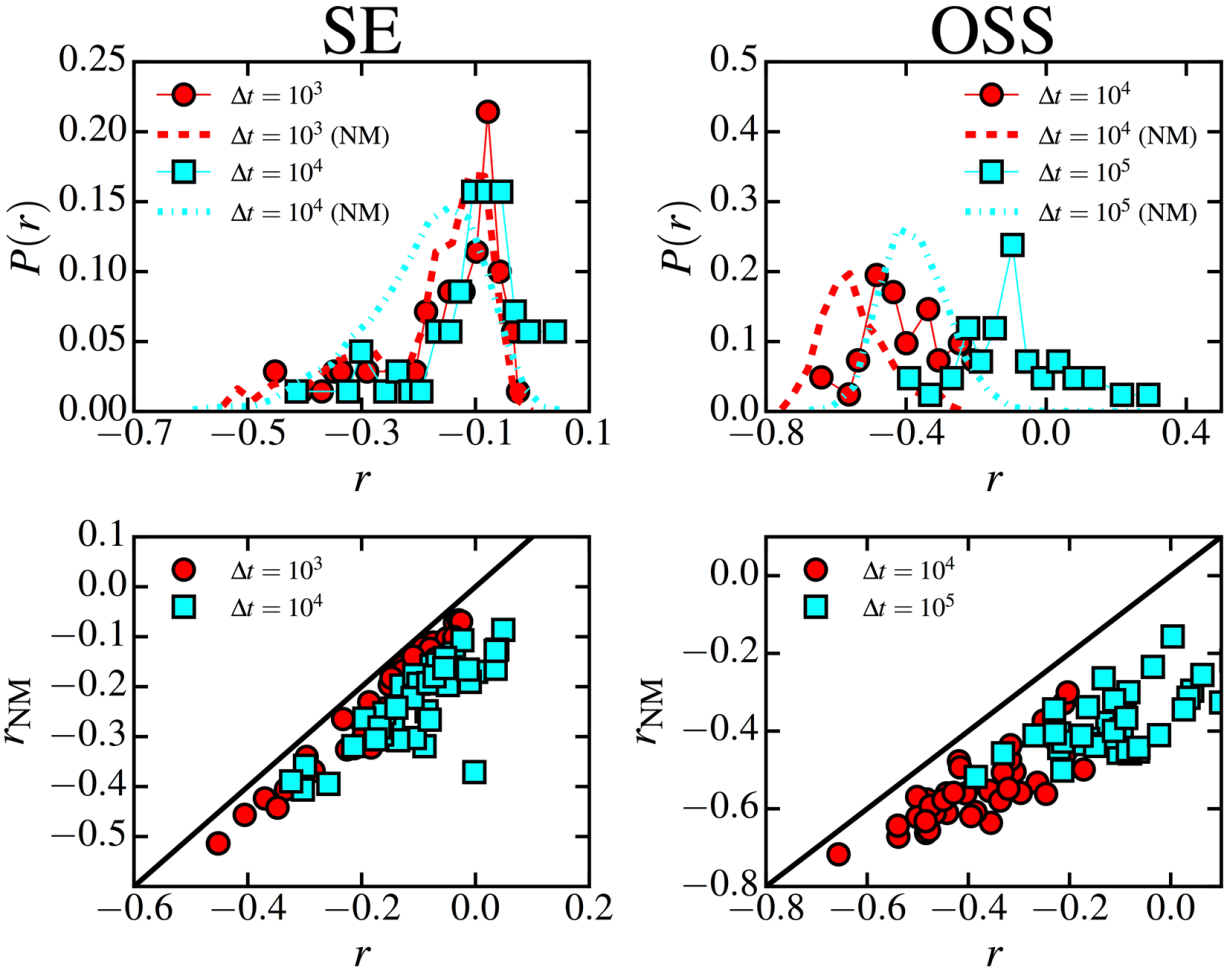
Comparison of the multitasking index of individuals in the original data, *r*, with the corresponding index *r*_*NM*_ in data randomized according the null model. Probability distribution of the multitasking index of the original and randomized data, *P*(*r*) and *P*(*r*_*NM*_) (top row), and scatter plot of the multitasking index of the original versus randomized data, *r* vs. *r*_NM_ (bottom row), for different time windows Δ*t*. In the scatter plots, only individuals with *r* with a p-value smaller than 0.05 or greater than 0.95 with respect to the null model are plotted. In calculating the multitasking index, we consider only individuals with at least 10 interactions in each layer. Data shown are: RM contact network, data set SE, 41 significant individuals over 73 (left, Δ*t* expressed in seconds) and OSS collaboration network, 40 significant individuals over 52 (right, Δ*t* expressed in minutes).

Figure 2 (top row) shows the probability distribution of the multitasking index, *P*(*r*), measured for each node of the SE contact and OSS collaboration networks (see Supplementary Fig. S3 for additional datasets), for different values of the time interval Δ*t*, obtained by cutting the whole temporal sequence into consecutive slices. The multitasking index is generally negative, indicating the presence of large sequences of uninterrupted acts of the same kind. In a given time interval Δ*t*, an individual is more likely to relate with the others only through face-to-face interactions, or only through calls or texts, and less likely to use both channels simultaneously. In the context of the OSS collaboration network, this translates into developers being more likely either to communicate or to co-work, not doing both actions at the same time. In APS networks, see Supplementary Fig. S3, it implies that authors are more likely to collaborate in a sequence of papers in the same journal, instead of switching among different journals.

This apparently strong effect must be, however, judged with caution, since burstiness alone is a sufficient condition for the emergence of large sequences of consecutive interactions in the same layer, even in temporally uncorrelated networks. In Section 4 of the Supplementary Material, indeed, we build a null model of an uncorrelated temporal multiplex network with two independent renewal processes, one for each layer $\ell$, each one with a power-law form of the inter-event time distribution, ${\psi }_{\ell }(\tau )\sim {\tau }^{-1-{\alpha }_{\ell }}$. We analytically show that the probability distribution of finding *n* consecutive events on the same layer $\ell$ follows a power-law form, ${P}_{\ell }(n) \sim {n}^{-(1+{\alpha }_{-\ell }/{\alpha }_{\ell })}$, see Supplementary Figs S5 and S6.

Therefore, the empirical multitasking index needs to be contrasted with a null model which destroys temporal correlations between layers. In this null model, for each individual *i*, the set all its interactions in each layer is randomized, in such a way that the interevent time distribution ${\psi }_{\ell }(\tau )$ of each layer $\ell$ is preserved, while temporal inter-layer correlations are destroyed. In Fig. 2, upper row, we show also the distribution of multitasking indexes in the randomized versions of our empirical datasets. As we can see, the real and randomized distributions are clearly different, specially for larger values of the time interval Δ*t*. In Fig. 2, bottom row (see also Supplementary Fig. S3), we present a scatter plot between the empirical and randomized coefficients, *r*(Δ*t*) and *r*_*NM*_(Δ*t*), respectively, for different time intervals Δ*t*. Only individuals whose coefficient *r*(Δ*t*) is significantly different from *r*_*NM*_(Δ*t*) (with a *p*-value smaller than 0.05 or greater than 0.95, see Section 2 of the Supplementary Material), are plotted.

One can see that almost all significant individuals have a multitasking coefficient *r*(Δ*t*) greater than the corresponding coefficient *r*_*NM*_(Δ*t*) obtained in the null model, as highlighted by the diagonal line. This implies that, in general, temporal inter-layer correlations tend to decrease the stretches of time in which activity is concentrated in a single layer, increasing the multitasking index. The practical implication of this observation for social dynamics is that temporal correlations increase the rate at which individuals switch from one kind of social activity to another one, with respect to a purely random behavior, only constrained by the burstiness of human dynamics.

### Effects of temporal correlations on coupled spreading dynamics

As we have seen, temporal correlations can alter the pattern of social interactions. Additionally, they can also influence the behavior of dynamical processes running on top of temporal multiplex networks. To show this, we consider the interplay of competing spreading processes, which has been previously studied on static, synthetic, multiplex networks^7, 25^. In this framework, an epidemic spreads on the physical layer of the RM contact networks while information spreads on the virtual layer, representing awareness to prevent the infection^25^. This scenario is modeled as follows: A Susceptible-Infected process runs on the physical layer, in which whenever an infected (*I*) individual *i* has a contact with a susceptible (*S*) one *j*, the disease is transmitted with probability *β*_1_, and *j* becomes infected. An Unaware-Aware process runs on the virtual layer, in which whenever an aware (*A*) individual *i* has a contact with an unaware (*U*) one *j*, the information is transmitted with probability *β*_2_, and *j* becomes aware. Infected individuals are instantaneously aware of the disease, while a susceptible individual that becomes aware of the disease is instantaneously immunized (*R*) from it, and cannot be infected.

**Figure 3:**
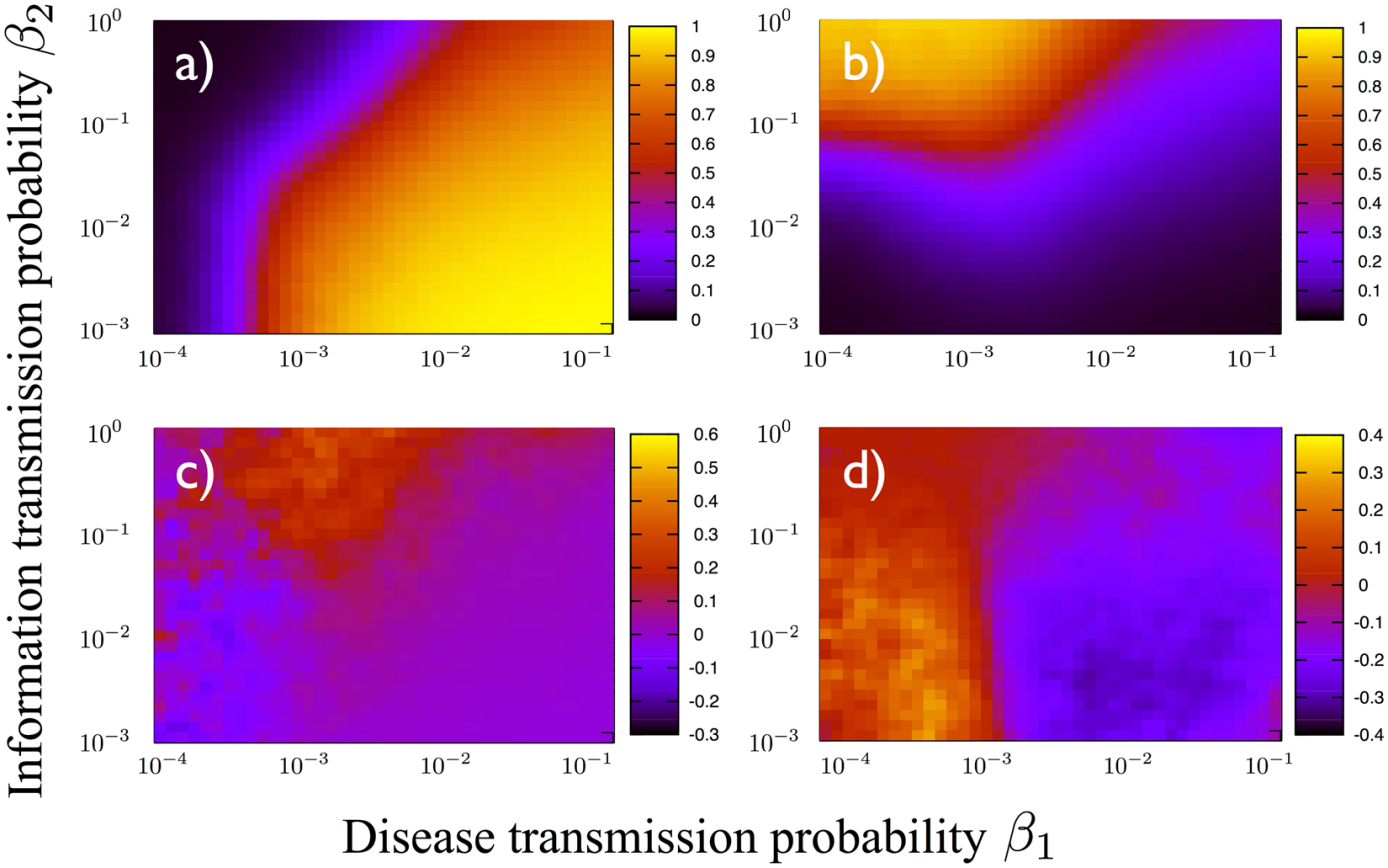
Phase diagrams (*β*_1_, *β*_2_) obtained by simulating the competition between epidemic spreading and information awareness on the RM contact network, data set SE (see Supplementary Fig. S4 for data set FF). Plots show (**a**) the fraction of infected and (**b**) fraction of immunized individuals for the original data; relative difference of infected (**c**) and immunized (**d**) individuals with respect to randomized data.

Figure 3 (top row) shows the final prevalence *ρ* = *I*_inf_/*N* (**a**) and the fraction of immunized individuals *i* = *R*_inf_/*N* (**b**) measured at the end of the contact sequence of data set SE (see also Section 3 of Supplementary Material and Supplementary Fig. S4 for data set FF), as a function of the two parameters *β*_1_ and *β*_2_ controlling the dynamics. The population shows a clear transition from an inactive (i.e. susceptible) to an active (i.e. infected) state, for increasing values of the infection probability *β*_1_, and decreasing values of the probability of information transmission *β*_2_. Interestingly, the fraction of immunized agents does not follow such behavior with respect to *β*_1_, but it reaches a maximum for ${\beta }_{1}\simeq {10}^{-3}$, and decreases for larger values. The effects of temporal correlations are shown in the bottom row of Fig. 3, where we plot the relative prevalence *ρ*_*R*_ = (*ρ*_NM_ − *ρ*)/*ρ*, (**c**) and relative fraction of immunized individuals, *i*_*R*_ = (*i*_NM_ − *i*)/*i*, (**d**), as obtained by contrasting original data with a null model (NM) in which the times of the sequence of contacts between any given pair of individuals *i* and *j* is randomized, destroying inter-layer temporal correlations while keeping the inter-event time distribution of contacts between pairs; see Section 2 of the Supplementary Material for further details.

The effect of temporal correlations on the epidemic outbreak is complex and nonlinear. On the one hand, the coupled spreading processes unfolding on a uncorrelated network result in a final prevalence up to 50% larger than the corresponding processes on a correlated multiplex. The maximum effect of temporal correlations on the prevalence is observed for large *β*_2_, close to the transition between the inactive and active phases. Therefore, temporal correlations slow down the epidemic spreading in these regions of the phase space, consequently reducing the disease outbreak. On the other hand, the final number of immunized individuals is larger in the uncorrelated case with respect to the correlated one for small *β*_1_, while it is smaller for large *β*_1_. This implies that temporal correlations slow down the information diffusion for small *β*_1_, and they speed it up for large *β*_1_.

## Discussion

Here we have shown that the presence of temporal correlations between the layers of a social multiplex networks can affect both the patterns of social contacts and the behavior of unfolding spreading processes. On the one hand, inter-layer correlations reduce the inclination of individuals to engage in large sequences of interactions of the same kind, as captured by the increase of their multitasking index. This observation means that individuals tend to switch from one kind of social activity to another one more frequently than would be expected in a purely random pattern of interactions. At the same level, this correlated pattern implies a certain degree of predictability in the sequence of contacts. On the other hand, temporal correlations alter the dynamics of coupled epidemic/awareness processes unfolding on different layers, either enhancing or depressing the spreading speed. In order to single out genuine temporal correlations between layers, in our analysis we contrast our results with appropriate null models, pointing out that the burstiness of human activity within a single layer is responsible for spurious correlations, and therefore it should be taken into account in the definition and measurement of new quantities related with social dynamics. Interestingly, the results obtained are independent of the length of the temporal sequence defining the multiplex, as evidenced by the SE and FF datasets, resulting from similar experiments but with widely different length.

Our study allows for a better understanding of social networks, highlighting the interplay between their twofold temporal and multi-layer nature, which allows to define and measure new observables able to characterize the entanglement in the development of different kinds of social activities. Moreover, our findings pave the way to sense and measure temporal correlations in other fields of complexity invested by the multiplex representation, ranging from the multilayer organization of brain networks^34^ to multimodal mobility and efficient transportation^35, 36^, as well as to their extension to more general multilayer networks. In particular, further research is in order to fully unravel the influence of inter-layer correlations in more complex epidemic spreading processes, as well as their impact on immunization strategies levering on the temporal patterns of social interactions^37, 38^.

## Materials and Methods

### Mathematical description of temporal multiplex networks

**Figure 4:**
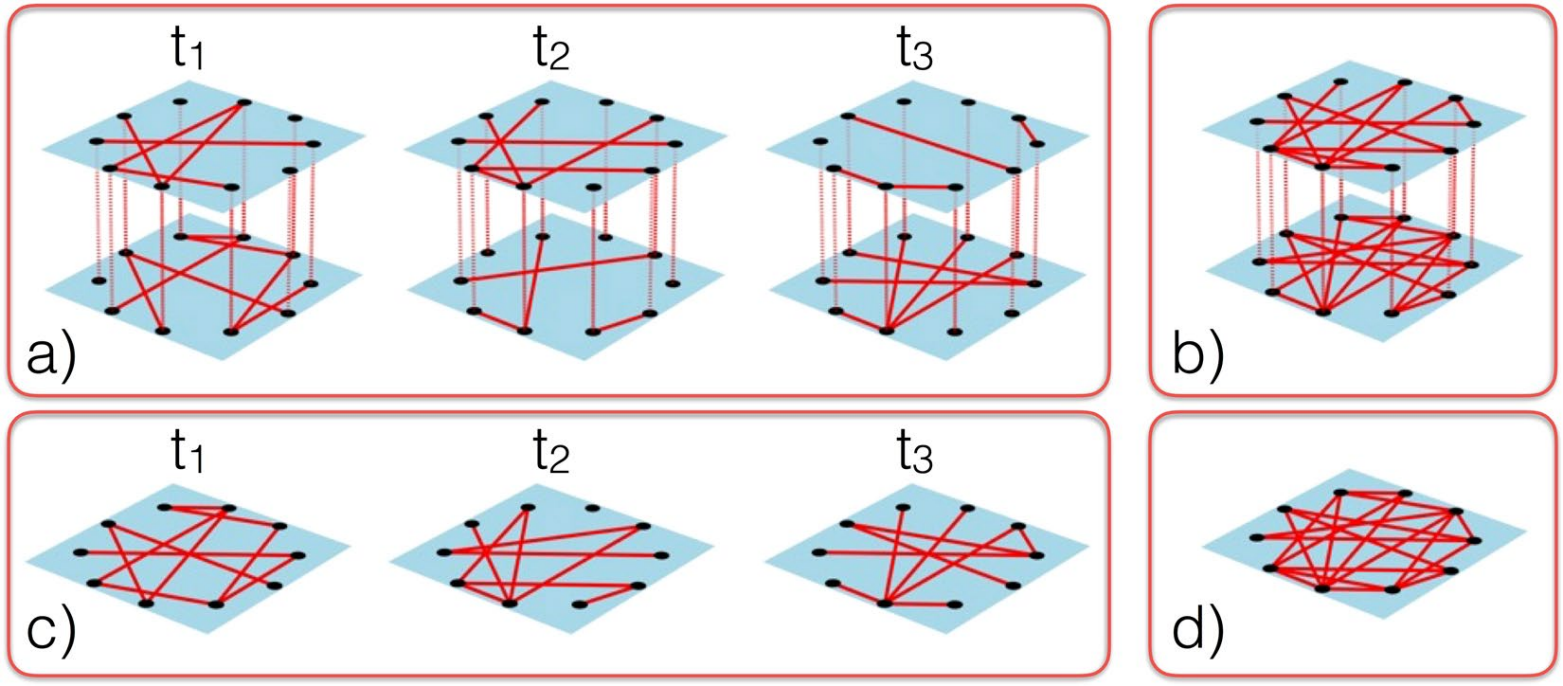
Different observation levels of a temporal multiplex network. A full temporal multiplex network (**a**), in this case with two levels, is represented by different snapshots at times ${t}_{i}\in {\mathscr{J}}=\{{t}_{1},{t}_{2},{t}_{3}\}$ of a single set of nodes with edges on different layers (colors) that appear at different times. The integrated static multiplex (**b**) is given by the projection over the time window ${\mathscr{J}}$ of all edges, which appear in their respective layers if they have appeared at least once in the whole observation window. A single layer temporal network (**c**) is obtained by projecting all layers onto a single one. Simultaneous projection over time and layers leads to a single layer static network (**d**).

Temporal multiplex networks can be mathematically described by endowing the multiplex paradigm with an additional temporal dimension^20^. In this way, a temporal multiplex network can be represented by a *contact sequence*, a set of quadruplets $(i,j,t,\ell )$ indicating that nodes *i* and *j* are connected at time *t* in layer $\ell$, with $i,j\in {\mathscr{V}}=\{\mathrm{1,}\ldots ,N\}$, the set of nodes, of a total number $|{\mathscr{V}}|=N$, $t\in {\mathscr{J}}$ the set of contact times, and $\ell \in  {\mathcal L} =\{{\ell }_{1},{\ell }_{2},\ldots ,{\ell }_{L}\}$, the set of $| {\mathcal L} |=L$ layers. From this exact description, coarse grained information can be obtained by projecting either temporal, multiplex or both dimensions onto a static and/or single-layered network, see Fig. 4. A single-layered temporal network is obtained by projecting different layers $\ell$ onto a single aggregate layer for each contact time $t\in {\mathscr{J}}$, so that the resulting temporal network is described in terms of a contact sequence with triplets (*i*, *j*, *t*). A static multiplex network is recovered by projecting time *t* onto a time-aggregated network for each layer $\ell$, resulting in a set of *L* (possibly weighted) networks, $\overrightarrow{G}=({G}_{{\ell }_{1}},{G}_{{\ell }_{2}},\ldots ,{G}_{{\ell }_{L}})$. Each network ${G}_{\ell }$ is described by the adjacency matrix^1^ ${{\bf{a}}}^{\ell }$, whose elements ${a}_{ij}^{\ell }={w}_{ij}^{\ell }={\sum }_{t}\chi (i,j,t,\ell )$ represent the number of interactions between *i* and *j* occurring over the whole contact sequence in layer $\ell$. One can project both time *t* and multiplexity $\ell$ onto a time-aggregated, single-layered network *G*. The elements of its adjacency matrix ${a}_{ij}={w}_{ij}={\sum }_{\ell ,t}\chi (i,j,t,\ell )$ represent the number of interactions between *i* and *j* occurring over the whole contact sequence across any layer $\ell$. The temporal dimension can also be considered in more general multi-layer networks^2^, in which each layer is characterized by a different set of nodes.

### Empirical data

We consider three different kinds of empirical temporal multiplex networks, all formed by two layers (duplex): human contact networks, recorded by the RM experiment^26^, OSS collaboration networks, reconstructed by means of data provided by the Apache software foundation^28^, and scientific collaboration networks, reconstructed from the APS data set for research^30^. The RM experiment^26^, conducted by the MIT Media Lab, is composed by two data sets: “Social Evolution” (SE) and “Friends and Family” (FF). It records proximity data by means of bluetooth sensors, forming a layer of physical interactions, $\ell =+1$, and digital communications, as given by phone calls and text messages, merged in a single layer of digital interactions $\ell =-1$. The Apache software foundation^28^ provides data of email communications between developers and their commits to edit files of several OSS project. We focus on “Apache Axis2/Java”, one of the project involving the largest number of developers, and consider a layer of co-work, $\ell =+1$, formed by co-commits to edit the same file, and a layer of communication, $\ell =-1$, formed by email messages. The APS dataset^30^ provides information about all papers published by the APS since 1893. A multiplex network can be constructed by considering the co-authorship of a paper published in any of the APS journals. We consider a layer formed by co-authorship in the journal Physical Review Letters (PRL), $\ell =+1$, and coauthorship in other APS journal, excluding PRL, $\ell =-1$.

### Null models of temporal multiplex networks

In order to single out inter-layer correlations in temporal multiplex networks, we consider different null models. From a theoretical point of view, the structure of a temporal multiplex network can be represented as a collection of point processes^39^ for each layer $\ell$, with two different levels of description:

1. A set of *N* point processes, ${\{{p}_{\ell ,i}\}}_{i\in {\mathscr{V}}}$, where ${\mathscr{V}}$ is the set of layers, in which a point corresponds to an interaction of an agent *i* with any other agent in the same layer;
2. A set of *N* ^2^ point processes, ${\{{p}_{\ell ,i,j}\}}_{i,j\in {\mathscr{V}}}$, in which a point corresponds to an interaction of agent *i* with agent *j* in the same layer.

The simplest characterization of these point processes is in terms of their inter-event time distributions representing the probability that two points in a process are separated by a time *τ*. Therefore, a null model of an uncorrelated temporal multiplex network corresponds to *N* × *L* (or *N* ^2^ × *L*) uncorrelated renewal processes^40^, depending on the level of coarse-graning one chooses to consider, in which the time *τ* between two points is an independent random variable distributed according to the inter-event time distribution *ψ*(*τ*) extracted from the data.

From an empirical point of view, null models can be constructed from the real data by a randomization processes^5^, in which interactions in each layer are reshuffled, preserving certain physical observables (mainly the inter-event time distributions). The null model for the multitasking index of individuals is based on the description level (1), preserving the individual inter-event time distributions, while case (2) has been used for the coupled spreading processes unfolding on the multiplex network, preserving now the inter-event time distributions of pairs of individuals. For the mutual information analysis, a rewiring preserving the uncorrelated entropy was performed. See Section 2 of the Supplementary Material for a detailed definition of each empirical null model.

## Electronic supplementary material

Supplementary Information
