# Supplementary material for: Effects of temporal correlations in social multiplex networks
Source: Sci Rep. 2017 Aug 17;7:8597. doi: 10.1038/s41598-017-07591-0 (PMC5561269; doi:10.1038/s41598-017-07591-0)
Supplement: Supplementary file 1 — Supplementary Information [file 41598_2017_7591_MOESM1_ESM.pdf]

Effects of temporal correlations in social multiplex networks: Supplementary Information

Michele Starnini[1,2], Andrea Baronchelli[3], and Romualdo Pastor-Satorras[4]

[1]Departament de Física Fonamental, Universitat de Barcelona, Martí i Franquès 1, 08028 Barcelona, Spain
[2]Universitat de Barcelona Institute of Complex Systems (UBICS), Universitat de Barcelona, Barcelona, Spain
[3]Department of Mathematics, City University London, Northampton Square, London EC1V 0HB, UK
[4]Departament de Física, Universitat Politècnica de Catalunya, Campus Nord B4, 08034 Barcelona, Spain

I. DETAILED DESCRIPTION OF THE EMPIRICAL DATASETS

A. RM contact network

The "Reality Mining" experiment provides two different data sets, both involving different groups of individuals interacting daily for a large period: "Friends and Family" (FF), involving a family residential adjacent to a university in the US, and "Social Evolution" (SE), performed on an undergraduate dormitory. Both provide data of three different kinds of social interactions: proximity or face-to-face (f2f) contacts, recorded by Bluetooth (BT) sensors, phone calls and text messages. Each data set is represented by a temporal duplex network, constituted by two different layers: a physical layer ($\ell = +1$), formed by f2f interactions, and a digital layer ($\ell = -1$), built by merging phone calls and text messages data. In order to reconstruct the multiplex network, we select only those individuals interacting in both layers.

Proximity interactions are recorded by BT technology every 5 minutes, while phone calls and text messages timing is recorded with precision of one second. We found that f2f interactions are not recorded exactly every 5 minutes, but there is a dispersion around this value. Therefore, we consider that a f2f contact between individuals i and j is interrupted if and only if the gap time between two consecutive records of an interactions between i and j is greater than 10 minutes. Since the resolutions of physical and digital contacts are considerably different (1 second versus 300 seconds or more), we decide not to aggregate interactions over an elementary time step. This choice ensures no data loss, given that aggregating calls and texts over a time window of 300 seconds (the proximity interactions time scale) would have lead to lost bursts of short-term interactions, a typical feature of the digital layer (e.g. bursts of text messages exchanged between a pair of individuals within a short time window). We consider the links formed by text messages as bidirectional, and neglect the temporal duration of interactions, representing social interactions as point-like events occurring at the first instant of their duration.

The main average properties of the FF and SE data sets are summarized in Table S1. FF and SE have a similar number of nodes N, and a very large duration T: Data set SE, in particular, covers the full academic year 2008/2009 and it is much longer than data set FF. Fig. S1 shows that the distribution of gap times τ between consecutive interactions of an individuals within the same layer ℓ, $\psi_\ell(\tau)$, is compatible with a power law, $\psi_\ell(\tau) \sim \tau^{-(1+\alpha_\ell)}$. The exponent α_ℓ is similar between physical ($\ell = +1$) and digital ($\ell = +1$) layer, $\alpha_{+1} = 1.0$ and $\alpha_{-1} = 0.7$, and notably it is the same between data sets FF and SE. Note that in the physical layer ($\ell = +1$) interevent gap times are larger than the minimum interval between consecutive interactions between the same pair, equal to 600 seconds. Since the large duration T, the aggregated multiplex network has a large average strength $\langle s\rangle = N^{-1}\sum_i s_i$, where the strength s_i of a node i is defined as the sum of the weights w_{ij} of the links to his neighbors $j \in \mathcal{N}_i$. This means that links have a large weight, i.e. each pair of individuals interact many times. The physical layer is much more dense than the digital one, and the overlap $O = E_{-1,+1}/\min\{E_{-1}, E_{+1}\}$ between them, where $E_{-1,+1}$ is the number of edges common to both layers [1], is very large, almost all links of the digital layer are present in the physical layer.

B. OSS collaboration network

We focus on the "Apache Axis2/Java", an open source software (OSS) project that is part of the Apache software foundation. [2] We reconstruct a duplex network by considering two layers, corresponding to co-commits by developers to the same code (work, $\ell = +1$), and email communications between them (talk, $\ell = -1$). In the OSS collaboration network nodes represent developers, connected by a link in layer $\ell = -1$ at time t_1 if they communicate by email at time t_1, and connected in layer $\ell = +1$ at time t_2 if co-commit to the same file, within a time-window of 24 hours, at time t_2. In order to reconstruct the multiplex network, we select only those developers who interacted at least once in the time window considered in both layers.

The main average properties of the OSS network are summarized in Table S1. The OSS network is the smallest network considered, with only 52 nodes, however, its large duration $T = 11$ years ensures a rich temporal pattern. Fig. S1 shows that the distribution of gap times τ between consecutive interactions of an individual within the same layer ℓ, $\psi_\ell(\tau)$, is compatible

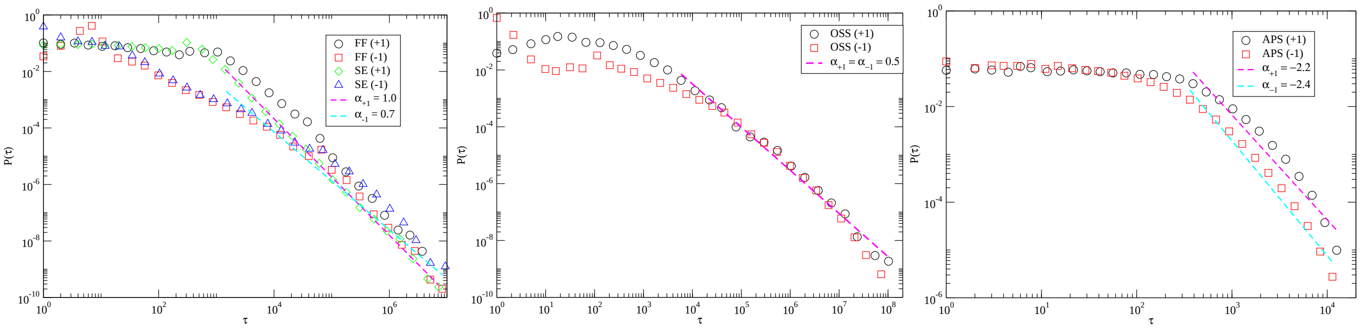

Figure S1: Interevent time distribution $\psi_\ell(\tau)$ of gap times between consecutive interactions of an individual in the same layer ℓ. Data shown are, from left to right: RM contact networks (expressed in seconds), data sets SE and FF, OSS collaboration network, and APS collaboration network (expressed in days). The interevent time distributions are compatible with power-law decay, $\psi_\ell(\tau) \sim \tau^{-(1+\alpha_\ell)}$, with an exponent depending on the data set considered, but similar between different layers. We note that the data sets FF and SE show a common behavior.

with a power law, $\psi_\ell(\tau) \sim \tau^{-(1+\alpha_\ell)}$. The exponent α_ℓ is the same between co-work ($\ell = +1$) and communication ($\ell = +1$) layer, $\alpha_{+1} = \alpha_{-1} = 0.5$. Since the large duration T, the aggregated multiplex network has a large average strength $\langle s \rangle$, which means that the links have a large weight, i.e. each pair of individuals interact many times. The communication layer is more dense than the co-work layer, and the overlap between them is very large, almost all links of the co-work layer are present in the communication layer.

C. APS collaboration network

The American Physical Society (APS) data sets for research [3] provide full information about all papers published in APS journals since 1893. From the APS data set, a duplex network is reconstructed by considering two layers, corresponding to co-authorship of a paper published in Physical Review Letters ($\ell = +1$), and a paper published in other APS journals ($\ell = -1$), namely in any of the followings: Physical Review A, Physical Review B, Physical Review C, Physical Review D, and Physical Review E. In order to reconstruct the multiplex network, we select only those authors who have at least a publication in both layers. In each layer $\ell = \pm 1$, two authors are thus connected by and edge at time t if they co-authored a paper published by APS, which has been received at time t. Since we consider as t the receiving date, the precision of the temporal interval is one day. In order to capture only actual social interactions in scientific collaboration, we select only papers with no more than 10 authors.

Table S1 summarizes the main properties of the APS network. The large duration T of the data, 52 years, from 1958 up to 2010, ensures a large number of authors, $N = 50077$. Fig. S1 shows the distribution of gap times τ between consecutive collaborations of a scientist in the same layer ℓ, $\psi_\ell(\tau)$. The decay of $\psi_\ell(\tau)$ for large τ is compatible with a power law form, $\psi_\ell(\tau) \sim \tau^{-(1+\alpha_\ell)}$, with similar exponents between different layers. Note that since we consider the date of the paper reception by APS, and not the publishing date, also very small intervals τ between consecutive papers are present. The aggregated multiplex network is

Interaction	Data set	Start date	N	T	O	Layer ℓ	E	$\langle k \rangle$	$\langle s \rangle$
Contact	SE	27/09/2008	73	242 d	255	Phys. $\ell = +1$	2422	66.4	9370
						Virt. $\ell = -1$	261	7.15	354
	FF	19/10/2010	74	156 d	122	Phys. $\ell = +1$	1958	52.9	1953
						Virt. $\ell = -1$	143	3.86	575
Collaboration	OSS	04/10/2001	52	11 y	237	Work $\ell = +1$	256	9.85	184
						Comm. $\ell = -1$	647	24.9	398
	APS	01/01/1958	50077	52 y	87204	PRL $\ell = +1$	171576	6.85	9.42
						nPRL $\ell = -1$	271851	10.9	20.6

Table S1: Some properties of the temporal multiplex networks under consideration: The RM contact networks, constituted by data sets FF and SE, and the OSS and APS collaboration networks. Properties shown are: Kind of social interaction (contact or collaboration); name of data set; starting date; N, number of nodes of the network; T, duration of the temporal network (in days for the contact networks and years for the collaboration network); O, overlap between layers in the aggregated multiplex; layer considered, $\ell = \pm 1$; E number of edges in each layer of the aggregated multiplex; $\langle k \rangle_\ell = N^{-1} \sum_i k_i^\ell$ average degree of each layer ℓ of the aggregated multiplex; $\langle s \rangle_\ell = N^{-1} \sum_{ij} w_{ij}^\ell$ average strength of each layer ℓ of the aggregated multiplex.

characterized by layers with similar densities, being the non-PRL layer denser and with larger average strength $\langle s \rangle$ than the PRL layer. The overlap O is quite large but is much smaller than in the contact networks, half of the links of the PRL layer being not present in the other layer.

II. NULL MODELS OF UNCORRELATED TEMPORAL MULTIPLEX NETWORKS

In order to check the statistical significance of the different observables measured in temporal multiplex networks, we have compared them with randomized versions obtained by applying different null models. We note the necessity to apply particularly tailored null models, since we want to preserve different properties of the multiplex time sequence to assess the effects of the different observables.

Here we describe in details the null models considered in the manuscript.

A. Entropy and mutual information between layers: Boostrapping analysis

In order to verify that the influence between layers found in the empirical networks is based on sufficient data, we perform a bootstrap analysis. We assume as null hypothesis that there is no influence between layers, i.e. the mutual information is equal to zero, and we estimated the probability (p-value) that the conditional entropy defined in Eq. 2 in the main paper is at least as low as the observed value. We perform a bootstrap resampling for each individual and we reject the null hypothesis if the p-value is smaller than 0.05.

The resampling procedure is defined in such a way as to keep constant the uncorrelated entropy defined in Eq. 1 in the main paper. To this end, for each individual i we select the set of pairs of consecutive interactions occurring in different layers, $\{(e^{\ell}_{j_1}, e^{-\ell}_{k_1}), (e^{\ell}_{j_2}, e^{-\ell}_{k_2}), \ldots, (e^{\ell}_{j_n}, e^{-}_{k_n}\ell)\}$, where $(e^{\ell}_{j}, e^{-\ell}_{k})$ is a pair of interactions of individual i with j in layer ℓ and with k in layer $-\ell$. To resample these pairs, we randomized the order of the set of second interactions occurring on layer $-\ell$, $\{e^{-\ell}_{k_1}, \ldots, e^{-\ell}_{k_n}\}$ and created random pairings with the set of first interactions occurring on layer ℓ, $\{e^{\ell}_{j_1}, \ldots, e^{\ell}_{j_n}\}$ thus destroying any temporal correlation between layers. After aggregating the transition probabilities for the random pairings, we calculated the conditional entropy for the randomized data. We repeated this procedure 200 times and calculate the p-value of each individual.

B. Randomized temporal multiplex network by preserving the interevent time distribution of single individuals

In order to evaluate the relevance of the multitasking index of individuals obtained in empirical networks, we compute these quantities on randomized networks. We build null models of a randomized temporal multiplex network in which we preserve the interevent time distribution $\psi_\ell(\tau)$ of each individual for each layer ℓ, while destroying temporal correlations between layers. We define a randomization procedure as follows: For each individual i, we swap all his interactions within the same layer, i.e. we consider the set of pairs $\{(j_1, t_1), (j_2, t_2), \ldots, (j_n, t_n)\}$, where a pair (j_i, t_i) represents an interaction with individual j_i at time t_i, we randomize the order of the individuals, $\{j_1, j_2, \ldots, j_n\}$, and create random pairings with the set of contact timing $\{t_1, t_2, \ldots, t_n\}$. This procedure ensures that the interevent time set $\tau = \{\tau_1, \ldots, \tau_{n-1}\}$, where $\tau_i = t_{i+1} - t_i$, is kept constant, and so it is the interevent time distribution $\psi(\tau)$. At the same time, temporal correlations between layers are washed out. We generate 200 bootstrap replicas for each empirical data set, and perform the analysis done for the original networks.

We calculate the multitasking coefficient of each individual in the rewired network, $r_{NM}(\Delta t)$, and verify the null hypothesis that this value is only due to the form of the interevent time distribution. We estimate the probability (p-value) that $r_{NM}(\Delta t)$ is as small or as large as the observed coefficient $r(\Delta t)$, and reject the null hypothesis if the p-value is smaller than 0.05 or larger than 0.95. The multitasking index is also computed on a synthetic temporal duplex network, $r_S(\Delta t)$, where each layer correspond to a temporal network generated independently using the Non-Poissonian activity driven (NoPAD) model [4], to ensure the lack of correlation between layers. We choose the same interevent time distribution $\psi_\ell(\tau)$ for the two layers, a power law distribution $\psi(\tau) = \alpha c(c\tau + 1)^{-1-\alpha}$, with $\alpha = 1.0$ and $c = 1.0$. Also the relevance of the probability distribution of consecutive interactions within the same layer, see Section IV, is controlled by contrasting the results with the same null model. We build the distribution of consecutive interactions obtained in the null model of rewired networks, $P^{NM}_{\ell}(n)$, for each layer ℓ, and compare with the original distribution $P_\ell(n)$.

C. Randomized temporal multiplex network by preserving the interevent time distribution of pairs of individuals

In order to evaluate the effects of the temporal correlations on the coupled spreading processes, we consider a null model that preserves the interevent time distribution for each pair of individuals in each layer separately, while destroying temporal

correlations between layers. The randomization procedure adopted here is similar to the one used for evaluating the significance of the multitasking index, but here we consider the set of point processes constituted by the interactions between pair of individuals. We notice, however, that the same procedure cannot be applied in the present context, since it can create spurious contacts between pairs of individuals. This effect is negligible for the computation of the multitasking coefficient, which is defined in terms of a single individual, while a spreading process involves explicit interactions between pairs.

The procedure is defined as follows: We consider all the interactions of each pair $i - j$ on the two layers, occurring at different times: $\mathcal{I}_{ij}^{\ell} = \{t_1^{\ell}, t_2 = t_1^{\ell} + \tau_1^{\ell}, \ldots, t_n^{\ell} = t_{n-1}^{\ell} + \tau_{n-1}^{\ell}\}$, where τ_n^{ℓ} is the gap between interaction n and interaction $n+1$ in layer ℓ. We then build new sequences, by randomizing the time order of the interevent time set of the two layers, $\mathcal{I}_{ij}^{\ell}$ and $\mathcal{I}_{ij}^{-\ell}$, but preserving the gaps $\{\tau_n^{\ell}\}$ for each layer. In this way the interevent time distribution of each pair $i - j$ in each layer ℓ, $\psi_{ij}^{\ell}(\tau)$, remains exactly the same. At the same time, temporal correlations between layers are washed out.

III. ADDITIONAL DATA SETS NOT SHOWN IN THE MAIN TEXT

A. Entropy and mutual information between layers

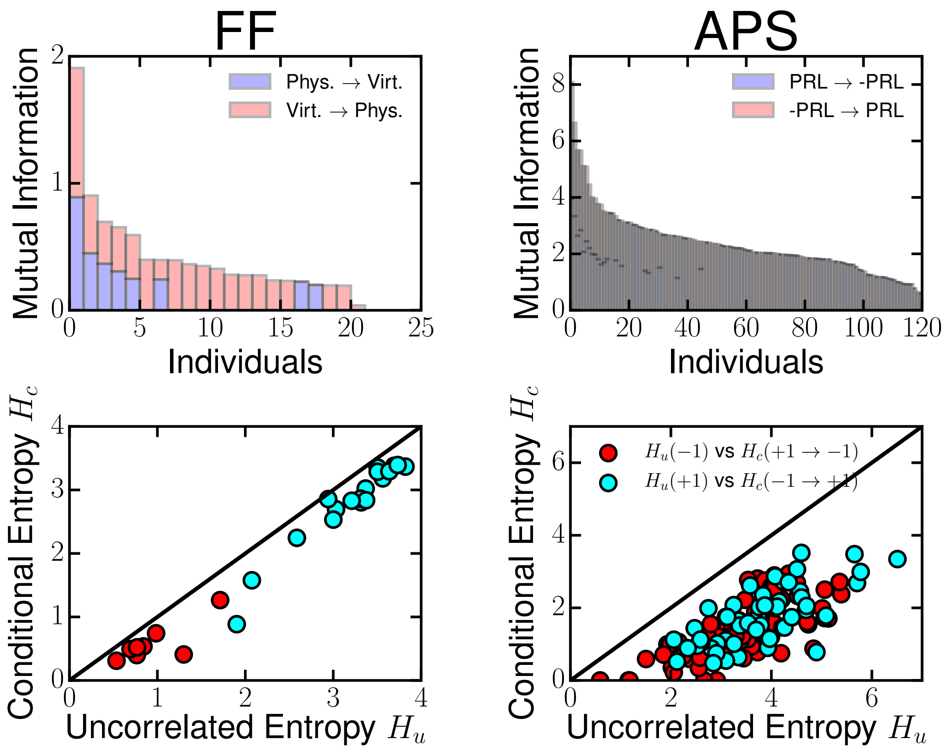

Figure S2: Entropy and mutual information between layers. Scatter plot of uncorrelated vs conditional entropy of each individual i, $H_i^u(\ell)$ vs $H_i^c(\ell \to -\ell)$ (bottom row), and mutual information between layers, $I_i(\ell \to -\ell)$ (top row). Only individuals with a conditional entropy with a p-value smaller than 0.05 with respect to the null model are plotted. Data shown are: RM contact network, data set FF (left) and APS collaboration network (right).

B. Multitasking index of individuals

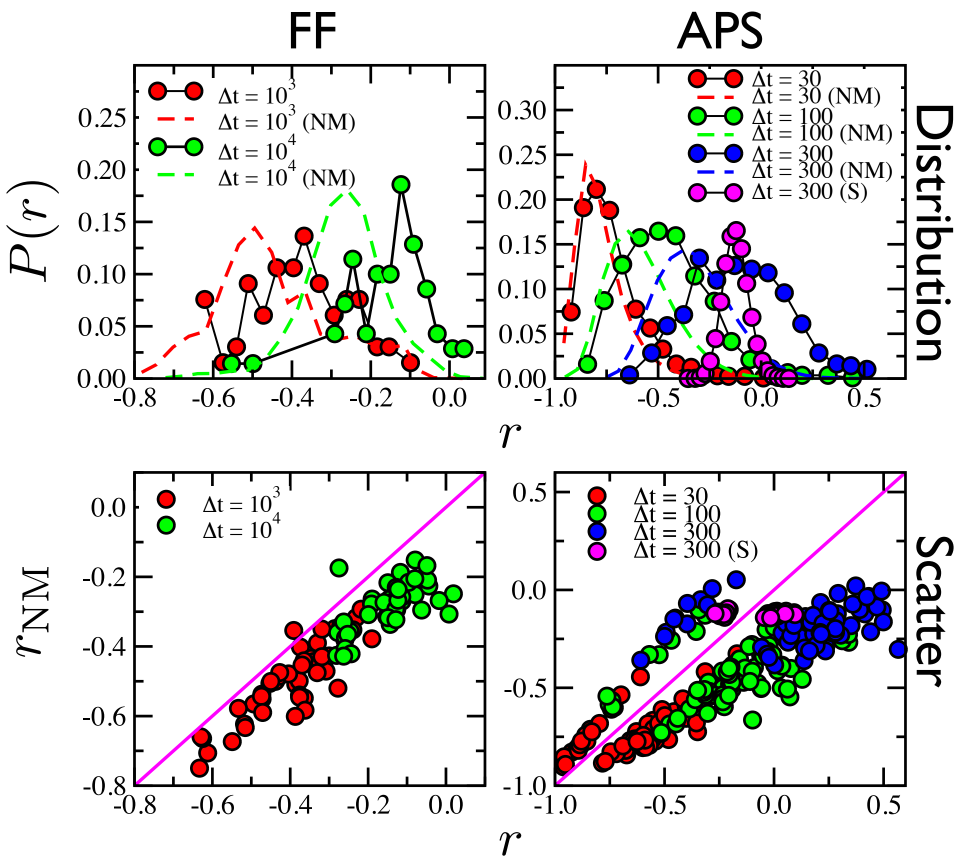

Figure S3: Probability distribution of the multitasking index of the original and randomized data, $P(r)$ and $P(r_{NM})$ (top row), and scatter plot of the multitasking index of the original versus randomized data, r vs. r_{NM} (bottom row), for different time window Δt and different data sets. In scatter plots, only individuals with r with a p-value smaller than 0.05 or greater than 0.95 with respect to the null model are plotted. In calculating the multitasking index, we consider only individuals with at least 10 interactions in each layer. Data shown are: RM contact networks, data set FF (left), with Δt expressed in seconds, and APS collaboration network (right), with Δt expressed in days. In the APS panels, we also plot the multitasking index of an uncorrelated synthetic network (labeled with S in plots) with the same number of nodes of the APS network.

C. Effects of temporal correlations of coupled spreading processes

In order to evaluate the effects of the temporal correlations on the coupled spreading processes, we run the processes on original and randomized data, and measure the relative difference of the prevalence ρ_R and the fraction of immunized individuals i_R, between them (see Main text). Each point of the phase space is averaged over 200 runs. Fig. S4 shows the results for the case of the FF data set. Results are similar to the ones shown for the SE data set. The final prevalence ρ (**a**) and the fraction of immunized individuals i_R (**b**) plotted in the phase space (β_1, β_2) show the same behavior of the SE data set. The effects of temporal correlations are weaker in this case, but nevertheless qualitatively similar. The final prevalence in the uncorrelated case is higher with respect to the correlated case for large β_2, close to the transition area, implying that temporal correlations reduce the epidemic outbreak for these values of the parameters (see Fig. S4 **c**). Also in this case the effect of temporal correlations on the final number of immunized individuals depends on the infection probability β_1: They slow down the information diffusion for small β_1, while speeding it up for large β_1. For this data set, however, the value at which the effect changes sign is larger than the other data set (see Fig. S4 **d**).

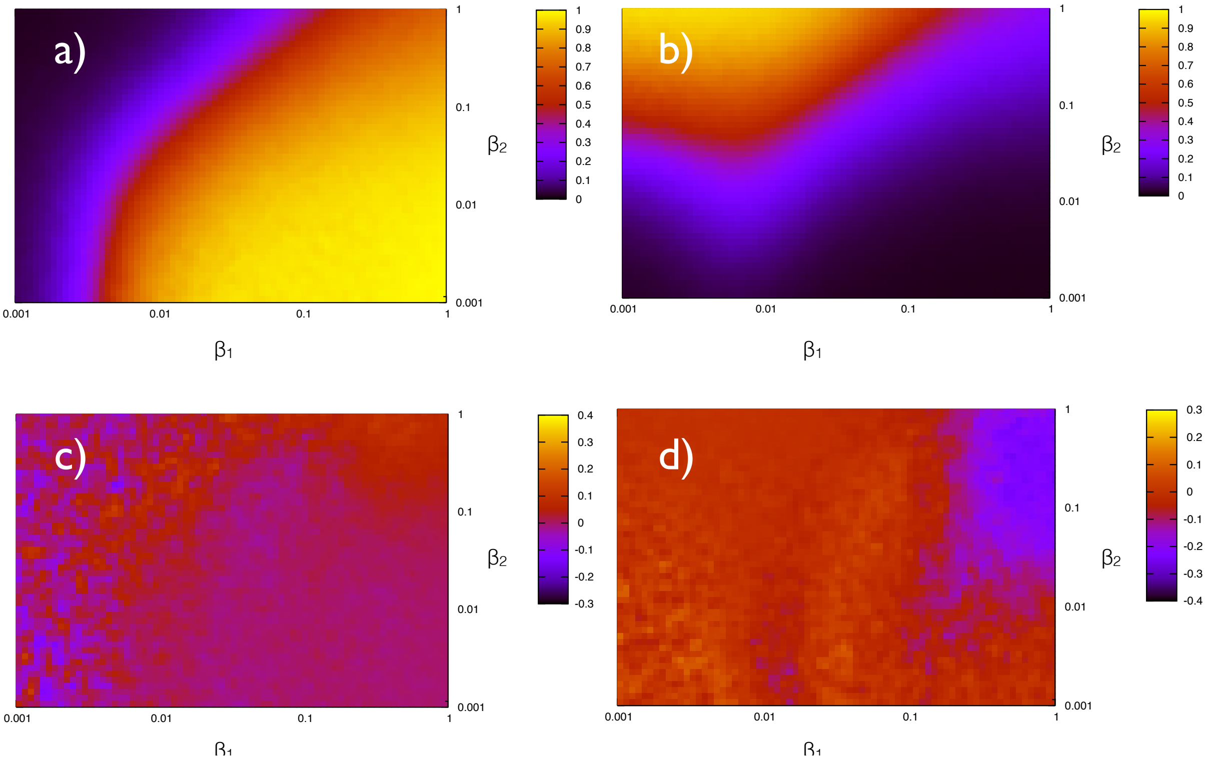

Figure S4: Effects of temporal correlations on coupled spreading processes. Phase diagrams (β_1, β_2) obtained by simulating the competition between epidemic spreading and information awareness on the FF data set. Top row shows the fraction of infected (**a**) and immunized (**b**) individuals for the original data, bottom row shows the relative difference of infected (**c**) and immunized (**d**) individuals with respect to randomized data.

IV. SEQUENCE OF CONSECUTIVE INTERACTIONS WITHIN THE SAME LAYER

The analysis of the multitasking index in empirical datasets shows the tendency of individuals to engage in long series of uninterrupted interactions in the same layer, a property that can be interpreted as an effect of temporal correlations. This same behavior can be also directly observed by computing the distribution $P_\ell(n)$ of the number n of consecutive events occurring within the same layer ℓ, not interrupted by any event occurring on other layers $\ell' \neq \ell$. Fig. S5 shows the empirical probability distribution $P_\ell(n)$ computed from all the data sets considered. The distributions are computed considering the interactions of a given individual i with any other individuals in the systems, and also for given pairs of individuals i and j. In all cases $P_\ell(n)$ is heavy tailed and compatible with a power law, $P_\ell(n) \sim n^{-\gamma}$. The exponent depends on considered data set, is slightly different between point processes constituted by individuals, γ_I, or pairs, γ_P (with the exception of APS networks, probably due to the scarcity of pairs of scientists with intense activity), and appears to be independent on the layer considered. The presence of uninterrupted sequences of consecutive interactions in the same layer might be interpreted as consequence of temporal correlations between layers, with interactions of one kind depressing interactions of the other kind. For example, it could be speculated that the tendency of an individual to relate with other peers through f2f contacts may reduce his probability to interact with them through calls or texts.

However, this interpretation does not take into account the role played by the burstiness of social interactions, since the presence in both layers of a broad tailed interevent time distribution, $\psi(\tau)$, represents a sufficient condition for the observed shape of $P_\ell(n)$, even if the layers are completely uncorrelated. Fig. S5, indeed, contrasts the form of the $P_\ell(n)$ obtained in the original data with the corresponding $P_\ell^{NM}(n)$ obtained in a null model which randomizes the multiplex networks by completely washing out temporal correlations between layers, but separately preserving the interevent time distribution $\psi_\ell(\tau)$ of each layer, see Section II C. Fig. S5 shows that there is no significant difference between original and randomized data, and indicates that the long tailed form of the distribution of consecutive interactions does not represent a signature of inter-layer correlations. Thus, the sequences of consecutive events of the same kind observed in the data is explained by the bursty nature of social acts.

Moreover, it is possible to show analytically that, taking as a null model two independent renewal processes [5], each one with a power-law form for the inter-event time distribution, $\psi_\ell(\tau) \sim \tau^{-1-\alpha_\ell}$, then the probability distribution of consecutive events of one series, not interrupted by any event of the other series, will follow a power-law form, $P_\ell(n) \sim n^{-1-\alpha_{-\ell}/\alpha_\ell}$. Let us consider

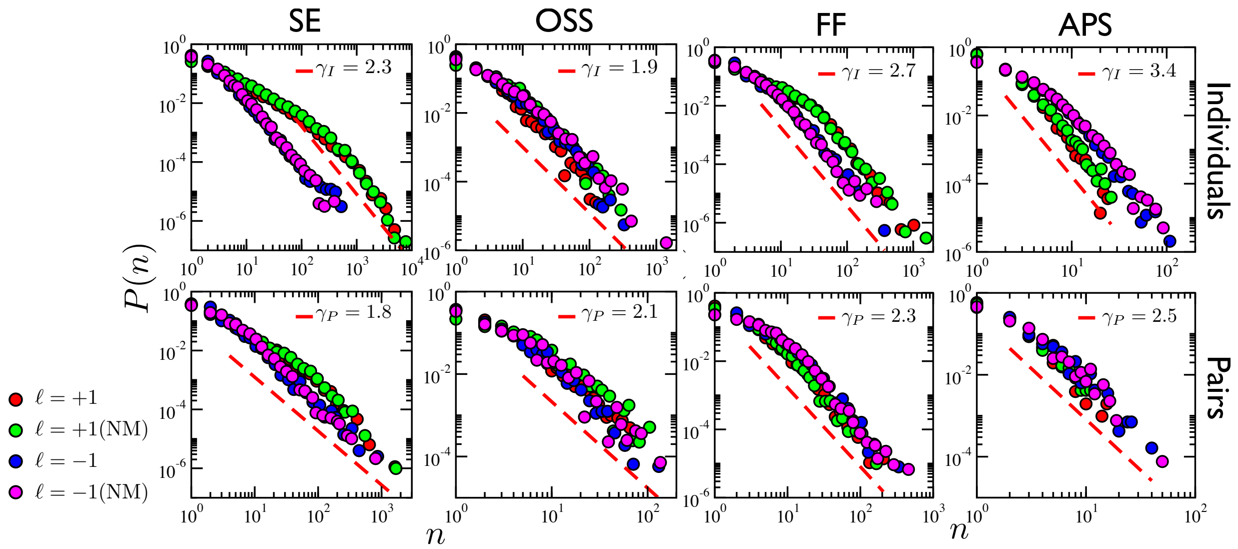

Figure S5: Probability of finding a number n of consecutive interactions occurring on the same layer ℓ, not interrupted by interactions on the other layer, $P_\ell(n)$, for different data sets considered and for the corresponding data randomized according the null model (NM). Probability distribution $P_\ell(n)$ for aggregated point processes constituted by the interactions of a single individuals (top panels) and a pair of individuals (bottom panels). Power-law decays, $P_\ell(n) \sim n^{-\gamma}$, with different exponent for pairs, γ_P, and individuals, γ_I, are plotted in dashed line. Data shown are, from left to right: RM contact networks, data set SE, and OSS collaboration network RM contact networks (data set FF) and APS collaboration network.

as a null model of uncorrelated temporal duplex network a multivariate point process formed by two renewal processes [5], one in each layer $\ell \in \{+1, -1\}$, with interevent time distributions $\psi_\ell(\tau)$.

We first consider, and as the simplest example, the scenario in which both layers obey a Poisson process[6] with rate λ_ℓ, i.e. $\psi_\ell(\tau) = \lambda_\ell e^{-\tau\lambda_\ell}$. Focusing on layer ℓ, assume two consecutive interactions in layer $-\ell$ at times $t_\star$ and $t_\star + \tau_\star$, with $\tau_\star$ a random number distributed according to $\psi_{-\ell}(\tau_\star)$. The number n of consecutive, uninterrupted interactions in layer ℓ in the interval $[t_\star, t_\star + \tau_\star]$ is given by a Poisson distribution[5] $P_\ell(n|\tau_\star) = (\lambda_\ell\tau_\star)^n e^{-\lambda_\ell\tau_\star}/n!$. Therefore, the probability of observing $n > 1$ consecutive interactions in layer ℓ is

$$P_\ell(n) = \int_0^\infty \psi_{-\ell}(\tau_\star)\frac{P_\ell(n|\tau_\star)}{\sum_{n'=1}^\infty P_\ell(n'|\tau_\star)}\, d\tau_\star = \frac{\lambda_{-\ell}}{\lambda_\ell}\zeta\left(n+1, \frac{\lambda_\ell + \lambda_{-\ell}}{\lambda_\ell}\right) \sim \left(\frac{\lambda_\ell}{\lambda_\ell + \lambda_{-\ell}}\right)^n, \tag{S1}$$

in the large n limit, where $\zeta(x, a)$ is the Riemann Zeta function. That is, $P_\ell(n)$ shows an exponential decay with a characteristic number of consecutive events $n_{\ell,c} = 1/\ln\left(\frac{\lambda_\ell+\lambda_{-\ell}}{\lambda_\ell}\right)$. The result in Eq. (S1) can be generalized for any interevent time distributions with finite first moment $\langle\tau\rangle_\ell$. In this case, the probability that a random interaction takes place in layer ℓ is $q_\ell = \langle\tau\rangle_\ell^{-1}/(\langle\tau\rangle_\ell^{-1} + \langle\tau\rangle_{-\ell}^{-1})$, and the probability of observing n consecutive interactions in layer ℓ is given by

$$P_\ell(n) \simeq (1-q_\ell)(q_\ell)^n = \frac{\langle\tau\rangle_{-\ell}^{-1}}{\langle\tau\rangle_\ell^{-1} + \langle\tau\rangle_{-\ell}^{-1}}\left(\frac{\langle\tau\rangle_\ell^{-1}}{\langle\tau\rangle_\ell^{-1} + \langle\tau\rangle_{-\ell}^{-1}}\right)^n, \tag{S2}$$

which can be mapped to Eq. (S1) by noting that, in Poisson processes, $\langle\tau\rangle = \lambda^{-1}$. The average number of consecutive events is in this case $\langle n\rangle_\ell = \langle\tau\rangle_{-\ell}/\langle\tau\rangle_\ell$, so that $\langle n\rangle_\ell = 1$ if the interevent time distribution of the two layers are equal.

Calculations in the Poissonian case are much simplified by the memoryless nature of these processes[6]. In the general case of non-Poissonian interevent time distributions, they become more involved, especially in the case of long tailed interevent time distributions, as those found in empirical temporal multiplex networks. Focusing again in layer ℓ, let us assume two consecutive events in layer $-\ell$, at times $t_\star$ and $t_\star + \tau_\star$. The number of events n in this interval in layer ℓ will depend also of the time of the last interaction in this layer, occurring at time $t_\ell < t_\star$, i.e. the number of events n depends on the aging time $t_a = t_\star - t_\ell$. The probability for the number of consecutive interactions in layer ℓ will then be given by

$$P_\ell(n) = \int_0^\infty \psi_{-\ell}(\tau_\star)P_\ell(n|t_a, \tau_\star)\, d\tau_\star, \tag{S3}$$

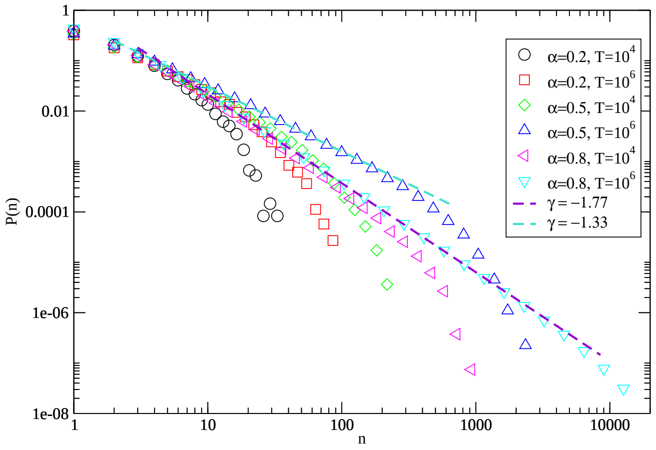

Figure S6: Sequence of consecutive interactions on the same layer in power-law distributed renewal processes. We plot the distribution $P_\ell(n)$ obtained from synthetic uncorrelated duplex networks represented by two uncorrelated renewal processes with the same interevent time distribution $\psi_\ell(\tau) \sim \tau^{-1-\alpha}$, for different values of α and different sampling windows T. The results depend non trivially on both α and T.

where $P_\ell(n|t_a, \tau_\star)$ is the probability of observing n renewal events in layer ℓ in the interval $[t_\star, t_\star + \tau_\star]$, knowing that the last renewal in ℓ took place at time $t_\ell = t_\star - t_a$. Analytic expressions for this function can be obtained in Laplace space[7]. They are however quite cumbersome, so, for the sake of simplicity, we will consider the non-aged case $t_a = 0$, in which we assume that the last interaction in layer ℓ happened simultaneously with the first interaction of the interval considered in layer $-\ell$, $t_\star = t_\ell$. Under this assumption, if we consider power-law forms of the interevent time distributions, $\psi_\ell(\tau) = \alpha_\ell c_\ell (c_\ell \tau + 1)^{-1-\alpha_\ell}$, where c_ℓ is some scale parameter, we can approximate[7], in the limit of large $n/(c_\ell \tau_\star)^{\alpha_\ell}$,

$$P_\ell(n|0, \tau_\star) \sim (c_\ell \tau_\star)^{-\alpha_\ell} e^{-A(n/\tau_\star^{\alpha_\ell})^{1/(1-\alpha_\ell)}}, \qquad \text{(S4)}$$

where A is a positive constant, depending on the parameters (c_ℓ, α_ℓ) of the interevent time distributions. From here, using Eq. (S3), we can obtain, within the non-aging approximation, the scaling form

$$P_\ell(n) \sim n^{-1-\alpha_{-\ell}/\alpha_\ell}, \qquad \text{(S5)}$$

In Supplementary Figure S6 we plot numerical results for $P_\ell(n)$ obtained from synthetic duplex networks with $\psi_\ell(\tau) \sim \tau^{-1-\alpha_\ell}$, for different values of α_ℓ and time windows T of observation. We observe that, while the $P_\ell(n)$ distributions are compatible with power-laws, the observed exponents do not quite match the theoretical prediction in Eq. (S5), depending in particular on the window length T. This fact is to be attributed to strong aging (memory) effects effects[7], which have been neglected in the derivation of the analytic prediction Eq. (S5).

---

[1] Menichetti, G., Remondini, D., Panzarasa, P., Mondragón, R. J. & Bianconi, G. Weighted multiplex networks. *CoRR* **abs/1312.6720** (2013).
[2] http://www.apache.org/. URL `http://www.apache.org/`.
[3] American Physical Society. Data sets for research. URL `https://publish.aps.org/datasets`.
[4] Moinet, A., Starnini, M. & Pastor-Satorras, R. Burstiness and Aging in Social Temporal Networks. *Phys. Rev. Lett.* **114**, 108701 (2015).
[5] Cox, D. R. *Renewal Theory* (Methuen, London, 1967).
[6] Kingman, J. *Poisson Processes*. Oxford Studies in Probability (Clarendon Press, Oxford, 1992).
[7] Schulz, J. H. P., Barkai, E. & Metzler, R. Aging Renewal Theory and Application to Random Walks. *Phys. Rev. X* **4**, 011028 (2014).
